# Integration of Art in Pathology Education: Insights from the Faculty of Medicine and Pharmacy of Agadir

**DOI:** 10.7759/cureus.79032

**Published:** 2025-02-15

**Authors:** Achref Miry, Mohammed Tbouda, Imad Chakri, Sanae Abbaoui

**Affiliations:** 1 Pathology, Faculty of Medicine and Pharmacy of Agadir, Agadir, MAR; 2 Pathology, Souss Massa University Hospital, Agadir, MAR; 3 Pathology, Oued Eddahab Military Hospital, Agadir, MAR; 4 Faculty of Medicine and Pharmacology, Ibn Zohr University, Agadir, MAR; 5 Epidemiology and Public Health, Faculty of Medicine and Pharmacy of Agadir, Agadir, MAR; 6 Epidemiology and Public Health, Souss Massa University Hospital, Agadir, MAR

**Keywords:** art, medical courses, medical education, medical students, pathology, practical session

## Abstract

Introduction

Traditional teaching methods in medical education, such as lectures and textbooks, may not always fully engage students, especially in pathology, where visual learning plays a crucial role. This study examines the effectiveness of using diagrams as a learning tool to improve comprehension in the general pathological anatomy module.

Materials and methods

At the Faculty of Medicine and Pharmacy of Agadir, 182 third-year medical students were divided into two groups. Group A observed and replicated simplified diagrams of three pathologies - acute appendicitis, nodal tuberculosis, and well-differentiated colonic adenocarcinoma - while Group B did not use diagrams. The effectiveness of this approach was evaluated through pre- and post-session questionnaires.

Results

The majority of students recognized drawing as a valuable learning tool, particularly for visually intensive medical modules. Although no significant difference was found between the experimental and control groups in their intent to use drawing in future studies (p = 0.158) or their perception of its effectiveness (p = 0.547), Group A showed a significant increase in willingness to incorporate drawing after the session (p < 0.01).

Conclusions

Visual learning, especially through diagram replication, can enhance pathology education. The significant increase in students’ willingness to incorporate drawing into their study methods highlights the potential value of integrating this approach into medical curricula.

## Introduction

In medicine, many disciplines rely on the interpretation of visual cues and the use of visual memory, both of which are fundamental to making accurate diagnoses and assimilating complex information for effective patient care. However, many medical students struggle to master the ability to recognize visual cues and translate them into meaningful clinical insights. This challenge is particularly evident in pathological anatomy, where visual memory and the interpretation of visual signs play a crucial role [1]. Developing these skills can contribute to the growth of visual intelligence, an essential cognitive ability for medical professionals [2].

Despite the importance of visual intelligence in medical education - especially in pathological anatomy - there are no standardized methods for systematically providing students with materials to cultivate these skills [3].

Over the past decade, the integration of drawing into medical education has demonstrated several benefits, including fostering tolerance for ambiguity and enhancing empathy [4-13]. Additionally, observational skills, a key competency in medical practice, have been significantly improved through the application of visual arts in medical training [5].

Artistic integration in medical education varies widely, ranging from passive approaches such as visits to medical museums to active methods like replicating or creating drawings of medical structures. Unfortunately, the latter approach has been less frequently implemented and reported, remaining largely limited to anatomy instruction [14-21].

The use of art and drawing in medicine dates back centuries, particularly in scientific research, where they were widely employed before the advent of photography. However, only in the past decade has their educational value been quantitatively assessed, revealing their role in enhancing learning in the medical sciences. The gradual incorporation of drawing into medical education has repeatedly shown benefits, including improved tolerance for ambiguity, increased empathy, and strengthened observational skills - critical components of effective clinical practice [4-13].

## Materials and methods

At the Faculty of Medicine and Pharmacy of Agadir, third-year students receive general pathology courses divided into four modules: inflammatory pathology, neoplastic pathology, vascular pathology, and storage diseases. The general pathological anatomy module serves as an introduction to specific pathological anatomy, where various neoplastic and nonneoplastic diseases are examined based on organ systems. Practical sessions are conducted to familiarize students with the diagnostic aspects of selected pathologies. For this study year, the chosen pathologies were acute appendicitis, tuberculosis, and well-differentiated colonic adenocarcinoma.

The practical sessions consist of two phases: a theoretical phase, where microphotographs illustrating key diagnostic features are presented, and a practical phase, where students analyze full virtual slides that mimic traditional microscopy. Examinations include questions on the interpretation of histological images.

Before the start of the general pathological anatomy practical sessions, 190 voluntary students were recruited and informed about the possible use of diagrams as learning aids, with instructions to bring drawing materials. These students were then divided into two groups. Group A received diagrams depicting the three selected pathologies, presented alongside microphotographs and notes describing the relevant morphological features for each diagnosis. Group B, in contrast, received only microphotographs and explanatory notes during the theoretical phase, which they later used to identify structures on virtual slides in the practical phase.

**Figure 1:**
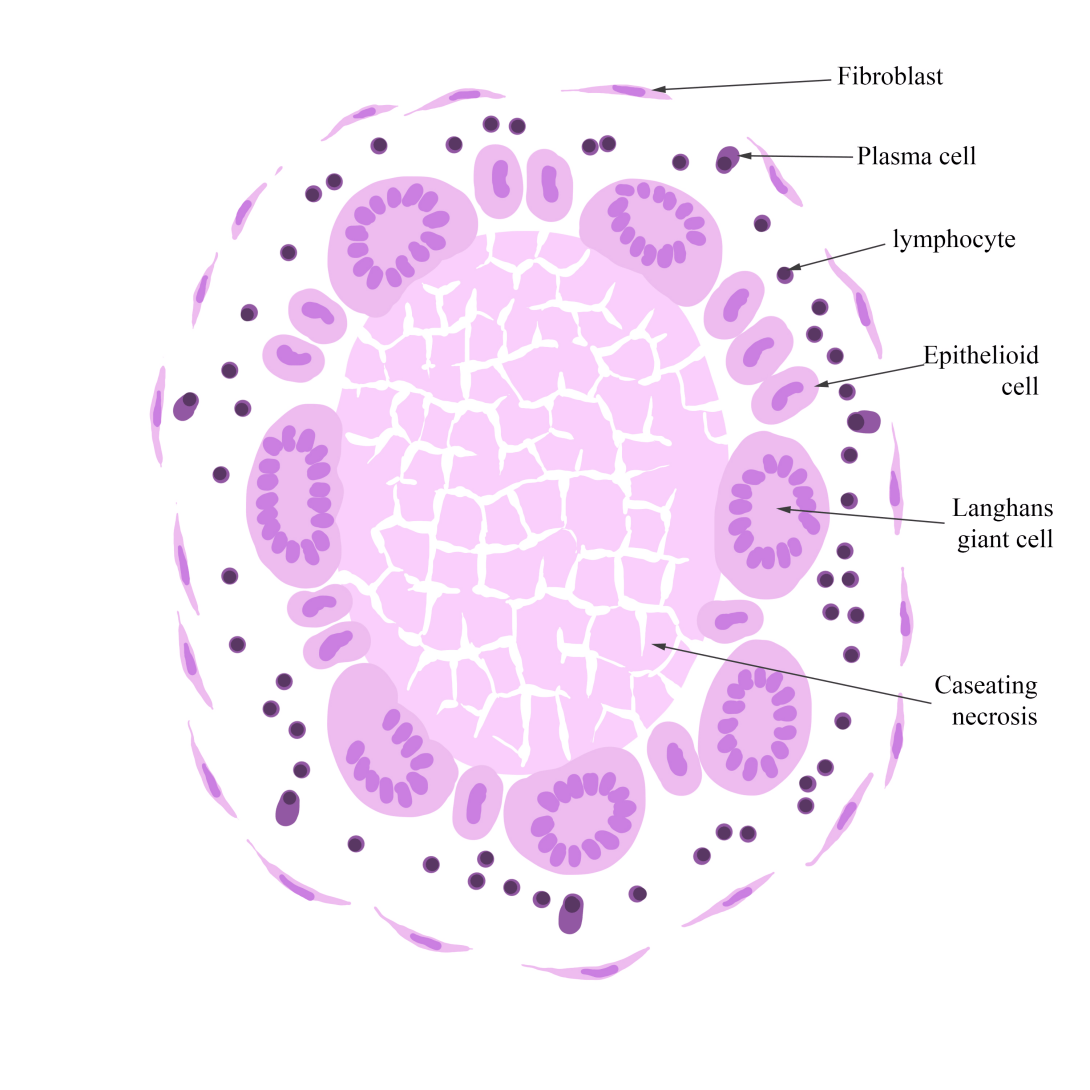
Diagram of a tuberculous granuloma presented to Group A during the practical phase of the lab session

The diagrams used in the sessions (Figure 1, Figure, Figure 3) were simple, employing minimal colors that resembled the staining patterns of H&E. They were designed to be easily replicated and featured comprehensive legends detailing the key morphological characteristics that students were expected to recognize and locate on virtual slides. After a brief explanation of the diagram components and legends, students in Group A reproduced the three diagrams as the theoretical session progressed, using either colored pencils or standard pencils. In contrast, students in Group B did not have access to any diagrams during their learning process.

**Figure 2:**
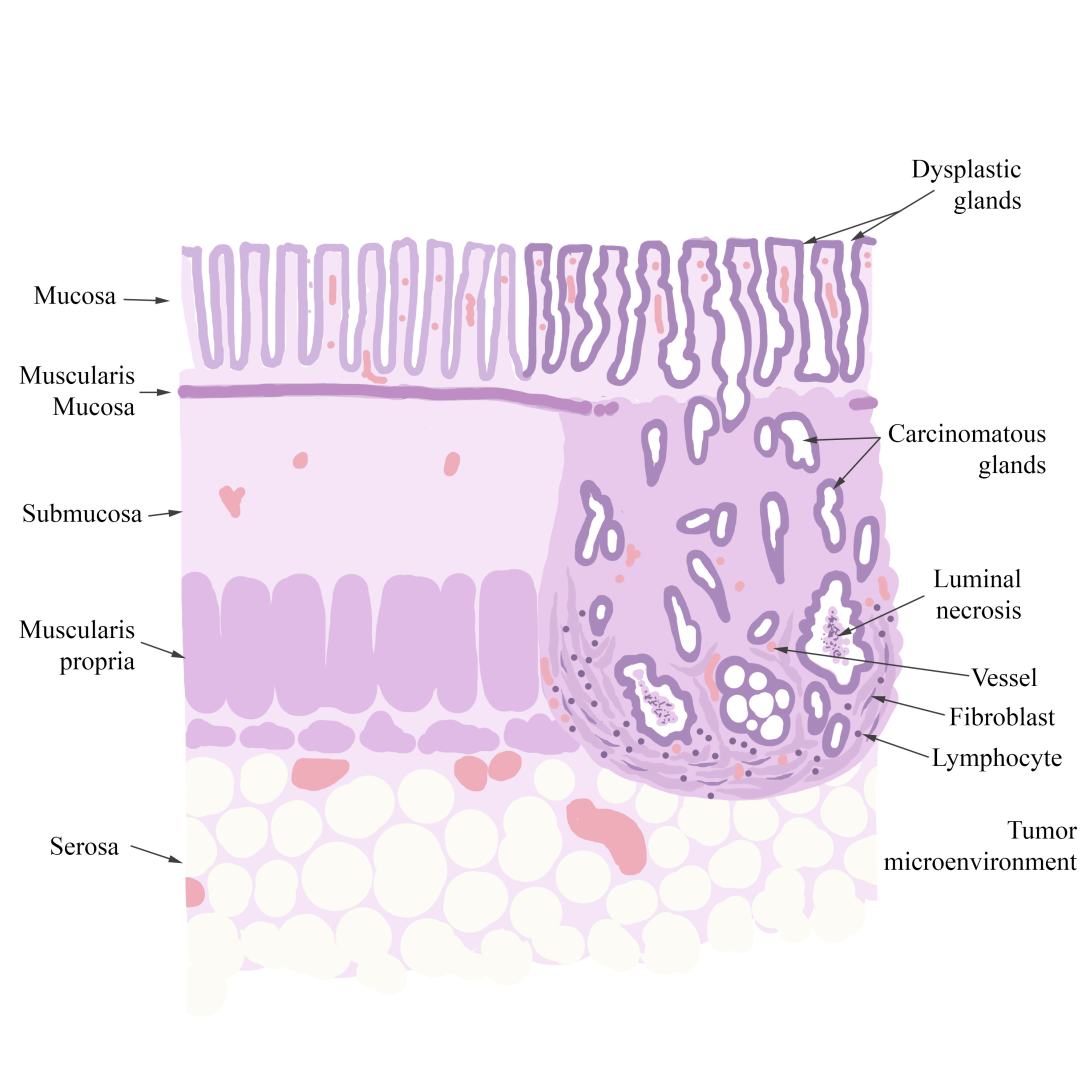
Diagram of well-differentiated colonic adenocarcinoma presented to Group A during the practical session

**Figure 3:**
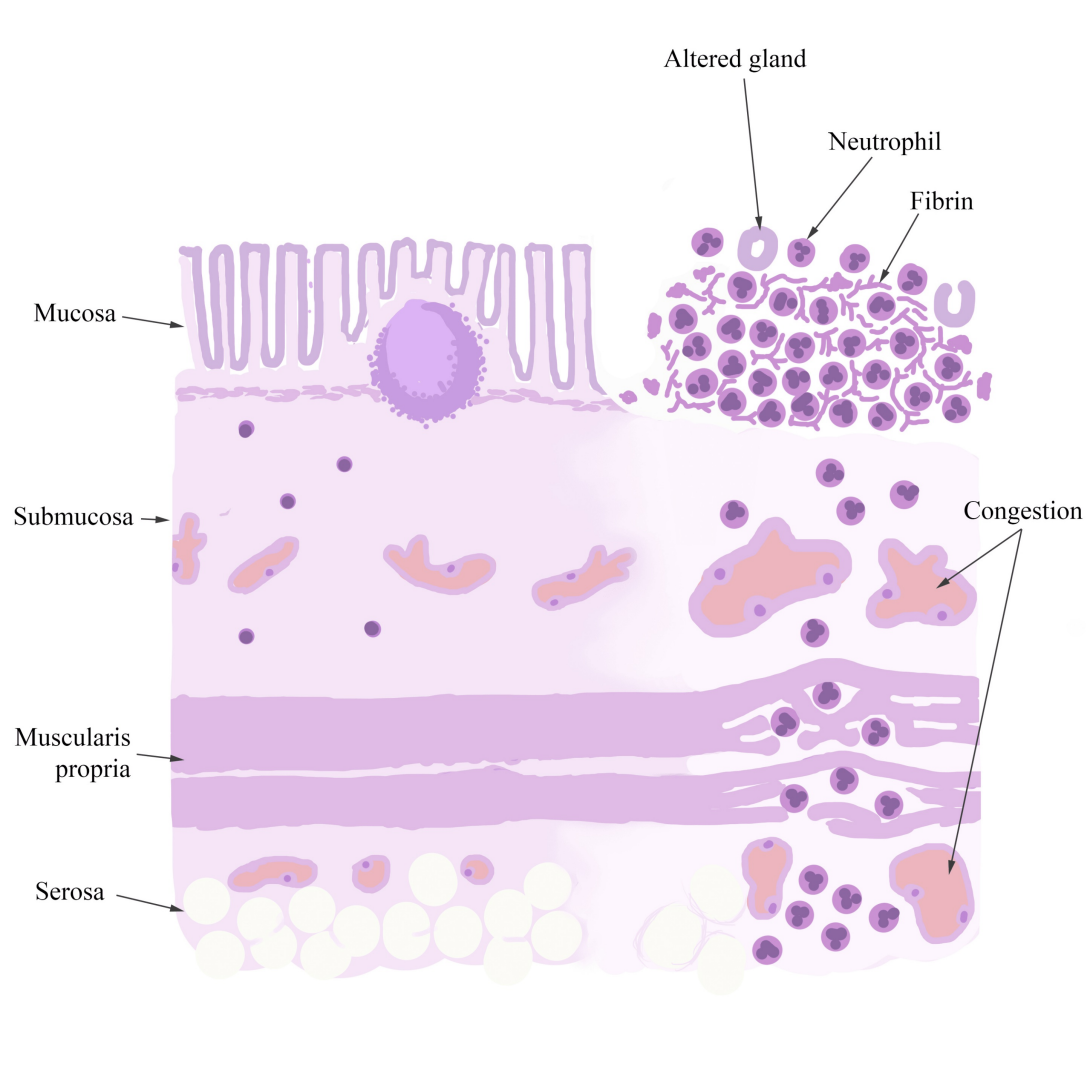
Diagram of acute appendicitis presented to Group A during the practical session

Questionnaires were administered before and after the practical sessions via QR codes scanned by students on their mobile phones. The purpose of these questionnaires was to assess students’ perceptions of drawing as a learning tool in medicine, particularly in pathological anatomy.

The pre-practical session questionnaire was distributed before the session began, while the post-practical session questionnaire was provided at the end. The pre-session questionnaire, identical for both Groups A and B, included questions on whether students considered drawing an effective tool for learning in medicine, with “yes” or “no” responses. It also asked whether they used drawing to study and review medical courses, with response options of “always,” “often,” “sometimes,” “rarely,” or “never.” Additionally, students who used drawing were asked in which discipline they applied it, allowing for short open-ended responses. Another question inquired whether having a passion for drawing was perceived as an advantage in learning medicine, with “yes” or “no” responses.

The post-practical session questionnaire differed slightly between the two groups. For Group A, it asked whether students considered drawing an effective learning tool in medicine and whether they planned to continue or start using drawing to study and review medical courses, with response options of “yes,” “no,” or “maybe.” The same questions were posed to Group B.

Statistical analysis was conducted using McNemar’s and chi-squared tests.

## Results

Survey results

A total of 182 third-year medical students volunteered to participate in the study and were randomly assigned into two groups: 100 students (52.63%) in the experimental group (Group A) and 82 students (47.37%) in the control group (Group B). All participants (100%) completed the questionnaire.

The results indicate that the majority of students, regardless of group assignment, considered drawing an effective tool for learning in medicine. Before the start of the practical sessions, 166 students (91.20%) expressed this view, while after the sessions, the proportion remained high at 89.73% (Table 1).

**Table 1:** Distribution of responses to the question “Do you find drawing to be an effective tool for learning in medicine?”

Responses to the first question	Number	Percentage (%)	Cumulative percentage (%)
No	16	8.80%	8.80%
Yes	166	91.20%	100%

A total of 140 students (78%) stated that having a passion for drawing is an advantage in learning medicine, while the remaining 40 students (22%) disagreed (Table 2).

**Table 2:** Distribution of responses to the question “Do you think having a passion for drawing is an advantage in learning medicine?”

Response to the second question	Number	Percentage (%)	Cumulative percentage
No	40	22%	22%
Yes	140	78%	100%

The frequency of drawing usage in different medical modules varied among students. A total of 3.29% reported using drawing permanently, 23.10% used it often, 46.15% sometimes, 20.90% rarely, and 6.56% never incorporated drawing into their medical studies (Table 3).

**Table 3:** Distribution of students based on the use of drawing

Frequency	Number	Percentage (%)	Cumulative percentage
Never	12	6.56%	6.60%
Sometimes	84	46.15%	50.70%
Rarely	38	20.90%	73.60%
Often	42	23.10%	96.70%
Always	6	3.29%	100%

Post-Term Survey Results

In the post-term survey, two comparisons were conducted: the first compared responses between Groups A and B, while the second examined changes within Group A before and after the practical session.

Comparison between Group A and Group B after the practical session

After the practical session, we compared groups A and B based on their responses to two questions. The first asked whether they planned to continue or start using drawing for medical studies, while the second inquired whether they considered drawing an effective learning tool in medicine. Analysis using contingency tables and the chi-squared test revealed no statistically significant differences between groups A and B. Table 4, Table 5, Table 6, and Table 7 present the results of these analyses.

**Table 4:** Contingency table between Group A and Group B after the practical session regarding the question “Do you plan to continue/start using drawing to study medicine?”

Response	Group A	Group B	Total
No (Observed % within a row)	11 (57.9%)	8 (42.1%)	19 (100.0%)
Yes (Observed % within a row)	48 (45.3%)	58 (54.7%)	106 (100.0%)
Perhaps (Observed % within a row)	36 (60.0%)	24 (40.0%)	60 (100.0%)
Total (Observed % within a row)	95 (51.4%)	90 (48.6%)	185 (100.0%)

**Table 5:** Chi-squared test results for the question “Do you plan to continue/start using drawing to study medicine?” df: degrees of freedom

Test statistic	Value	Df	p-value
Chi-squared	3.68	2	0.158
N	185		

**Table 6:** Contingency table between Group A and Group B after the practical session regarding the question “Do you think that drawing is an effective tool for learning in medicine?”

Response	Group A	Group B	Total
No (Observed % within a row)	11 (57.9%)	8 (42.1%)	19 (100.0%)
Yes (Observed % within a row)	84 (50.6 %)	82 (49.4 %)	166 (100.0%)
Total (Observed % within a row)	95 (51.4%)	90 (48.6%)	185 (100.0%)

**Table 7:** Chi-squared test results df: degrees of freedom

Test statistic	Value	df	p-value
Chi-squared	0.363	1	0.547
N	185		

A comparable proportion of students in both the experimental and control groups indicated their intention to continue or start using drawing for medical studies (45.3% vs. 54.7%; p = 0.158) (Table 4).

Likewise, similar proportions of students in both groups reported that they viewed drawing as an effective tool for learning in medicine (50.6% vs. 49.4%; p = 0.547) (Table 6).

Comparison within Group A before and after the practical session

In our study, we also analyzed changes within Group A by comparing responses to the same two questions before and after the practical session. This within-group comparison aimed to assess whether participation in the session influenced students’ perspectives.

The results of the contingency analysis and McNemar’s test are presented in Table 8, Table 9, Table 10, and Table 11.

**Table 8:** Contingency table for Group A before and after the practical session regarding the question “Do you plan to continue/start using drawing to study medicine?”

Response	No	Yes	Total
No (Observed % within a row)	2 (28.6 %)	5 (71.4%)	7
Yes (Observed % within a row)	9 (10.2%)	79 (89.8%)	88
Total (Observed % within a row)	11 (11.6%)	84 (88.4%)	95

**Table 9:** McNemar’s test results df: degrees of freedom

Test statistic	Value	df	p-value
Chi-squared	1.14	1	0.285
N	95		

**Table 10:** Contingency table for Group A before and after the practical session on the question “Do you plan to continue/start using drawing?”

Response	No	Yes	Total
No (Observed % within a row)	2 (28.6%)	5 (71.4%)	7
Yes (Observed % within a row)	9 (10.2%)	79 (89.8%)	88
Total (Observed % within a row)	11 (11.6%)	8 (88.4%)	95

**Table 11:** McNemar’s test results df: degrees of freedom

Test statistic	Value	df	p-value
Chi-squared	60.2	1	<0.01
N	95		

Contingency analysis of responses to the question “Do you plan to continue/start using drawing?” reveals a statistically significant difference in Group A’s answers before versus after the practical sessions (p < 0.01; Table 10, Table 11).

## Discussion

Our study is one of the few, if not the only one, to explore the application of drawing and visual art in teaching pathological anatomy. Nevertheless, this work may inspire educators in other medical disciplines to incorporate drawing into their teaching methods. While previous studies have examined the role of drawing in medical education, most have focused on histology [14,22-25].

The objective of our study was to assess students’ perceptions of using drawing as a learning tool in pathological anatomy and to evaluate the impact of this approach on their future engagement with drawing. Our findings indicate that most students perceive drawing as an effective and valuable method for learning medicine, particularly for subjects that rely heavily on visual memory. However, the frequency of drawing use varied across different medical modules. A post-session survey comparing responses within Group A before and after the practical session showed a statistically significant difference in responses to the question “Do you plan to continue/start using drawing?” (p < 0.01).

A similar study investigating students’ perceptions of drawing in histology education was conducted among first-year medical students who were encouraged to draw histological images [22]. In that study, nearly half of the participants (47.5%) reported plans to use drawing in the future to study various medical disciplines. Furthermore, students in the experimental group who incorporated drawing into their histology sessions demonstrated a more favorable response than those who attended conventional sessions without drawing (75% vs. 40.6%; p = 0.10) [22].

This same study highlighted that a significant number of students foresee integrating drawing into their medical studies and support its inclusion in medical curricula. Beyond histology and pathological anatomy, research exploring medical students’ perspectives on drawing has consistently reported positive attitudes toward its use [4,22,26].

Unlike a study conducted in the United States, where first-year medical students were invited to voluntarily reproduce histological diagrams, our approach involved providing simple, faculty-approved diagrams specific to pathological anatomy. Students were then asked to replicate these diagrams as accurately as possible [22]. We believe that the voluntary nature of drawing in that study may have been limited by the complexity of histological structures, as students likely opted for simpler diagrams.

There are certain limitations to consider in our study. Verifying the accuracy of student-reproduced diagrams was challenging, particularly given the large number of participants. Additionally, student motivation for drawing may have been influenced by the incentive of a raffle for those who submitted their diagrams. However, our primary goal was to evaluate student reception toward integrating diagrams into pathological anatomy teaching, whether in practical sessions or lectures. In the future, reproducing diagrams could even be incorporated into exam assessments.

Students who participated in the drawing activity were able to produce sufficiently representative diagrams using colored pencils or, in some cases, regular pencils (Figure 2). They brought their own materials after being informed in advance about the possibility of drawing during the practical session.

Our proposed approach promotes active student engagement, particularly as pathological anatomy, like histology, is becoming increasingly digitized. There is a risk that teaching these subjects could shift toward passive observation rather than interactive learning [27]. Drawing allows students to understand and identify key microscopic features of specific entities. Compared to real microphotographs, simplified drawings exclude excessive detail that may not be essential for a third-year medical student [28]. In contrast, microphotographs often contain a high level of detail, which could hinder the learning process [28]. Another advantage of drawing, as noted in some publications, is that it provides a refreshing break from traditional didactic methods [28].

Beyond drawing, other active learning strategies - such as virtual slides, microphotography, small-group activities, and interactive quizzes - have been reported in the literature as effective tools for student engagement in histology [29,30]. Additionally, unconventional approaches have also been explored, such as body self-painting for anatomy learning, which was highlighted in a recent study [30].

Future research will be conducted to assess the long-term impact of integrating diagrams into pathological anatomy education, particularly in terms of knowledge retention. Additionally, we aim to investigate the proportion of students who incorporate drawing into other medical subjects.

## Conclusions

In our study, the majority of medical students regarded art and drawing as effective and valuable learning methods. We observed a strong interest in drawing during pathology practical sessions, with many students indicating they would apply these techniques to other educational modules. We believe that integrating diagrams can serve as a complementary tool to enhance pathology education.
